# Spatio–temporal hotspots of satellite–tracked arctic foxes reveal a large detection range in a mammalian predator

**DOI:** 10.1186/s40462-015-0065-2

**Published:** 2015-11-15

**Authors:** Sandra Lai, Joël Bêty, Dominique Berteaux

**Affiliations:** Canada Research Chair on Northern Biodiversity, Centre for Northern Studies and Quebec Center for Biodiversity Science, Université du Québec à Rimouski, 300 Allée des Ursulines, Rimouski, QC G5L 3A1 Canada

**Keywords:** Argos satellite tracking, *Vulpes lagopus*, Sea ice, Spatio–temporal hotspots, Detection range, Scavenging, Dynamic Brownian bridge movement model

## Abstract

**Background:**

The scale at which animals perceive their environment is a strong fitness determinant, yet few empirical estimates of animal detection ranges exist, especially in mammalian predators. Using daily Argos satellite tracking of 26 adult arctic foxes (*Vulpes lagopus*) during a single winter in the High Canadian Arctic, we investigated the detection range of arctic foxes by detecting hotspots of fox activity on the sea ice.

**Results:**

While maintaining territories in the tundra, these solitary foragers occasionally used the sea ice where they sometimes formed spatio–temporal hotspots, likely scavenging on marine mammal carcasses. We detected 35 movements by 13 individuals forming five hotspots. Foxes often traveled more than 10 km, and up to 40 km, to reach hotspots, which lasted one–two weeks and could gather up to 12 individuals. The likelihood of a fox joining a hotspot was neither influenced by its distance from the hotspot nor by the distance of its home range to the coast.

**Conclusions:**

Observed traveling distances may indicate a high detection range in arctic foxes, and our results suggest their ability to detect food sources on the sea ice from their terrestrial home range. While revealing a wide knowledge gap regarding resource detection abilities in mammalian predators, our study provides estimates of detection range useful for interpreting and modeling animal movements. It also allows a better understanding of foraging behavior and navigation capacity in terrestrial predators.

**Electronic supplementary material:**

The online version of this article (doi:10.1186/s40462-015-0065-2) contains supplementary material, which is available to authorized users.

## Background

The scale at which animals perceive their environment determines their ability to locate resources and avoid predators [1, 2], and is thus a key ingredient of individual fitness. Accordingly, it is central to a broad range of ecological fields, including behavioral ecology, movement ecology, landscape ecology and evolutionary ecology [1–4]. This information is for example critical when modeling animal movements, especially in information–based approaches, where an animal’s decisions need to be set according to its perceptual range or sensory abilities [3–5]. However, because it is very difficult to estimate, there are few empirical measures of the distance over which animals can assess their environment [1, 6, 7]. The detection range of a species, defined here as the distance over which individuals can discover a resource [7, 8], involves sensory abilities, movement capacities, as well as social foraging tactics enabling information transfer about resource locations [8–12]. Empirical measurement of detection ranges usually relies on the visual observation of animals [7, 13, 14] or their electronic tracking coupled with an assessment of resource acquisition [15, 16].

Scavengers should be excellent study models to analyze detection range of animals, for they need to locate carrion, a spatially and temporally aggregated resource pulse that can be readily identified by observers. Yet, whereas many studies have focused on the organization of scavenger guilds [12, 17–20], little work has been done on the ability of individuals to scavenge [21]. Carrion can attract and concentrate high numbers of consumers, whether they are of local or distant origin [8]. High carrion detection performance is attributed to birds like ravens (*Corvus corax*) and bald eagles (*Haliaeetus leucocephalus*) [8, 17], which have an excellent vision [22], can cover large distances at little costs [23] and also benefit from social information transfer [9, 11]. Mammals are usually considered to be less efficient than birds at locating carrion [8, 23].

Arctic foxes (*Vulpes lagopus*) are facultative scavengers that can feed on marine carrion found on the sea ice during winter [24–26]. They can gather in great numbers around marine mammal carcasses [24], have a good sense of smell [27, 28], are able to cover large distances at a fast pace [29], and therefore provide an opportunity to investigate animal detection abilities. From an ecosystem perspective, arctic foxes moving from the land to scavenge on the sea ice can function as active mobile links and resource linkers [30], enhancing the connectivity and energy transfers between the marine and terrestrial ecosystems [25, 31]. Considering the risks and energetic costs of searching resources outside of the usual home range, their ability to detect food in an unfamiliar environment such as the sea ice may influence their foraging decisions and thus their movement patterns, which may in turn influence the flow of nutrients from the sea to the tundra. Therefore, it is important to investigate the detection range of such mobile species. Here, we show through satellite tracking that arctic foxes foraging on the sea ice can reveal an unexpectedly long–distance detection range in a mammalian scavenger.

We answer two specific objectives. First, using the tendency of foxes to gather near carrion, we locate areas intensively used by foxes on the sea ice and identify the number of individuals at these spatio–temporal hotspots, the distances traveled by foxes to reach them, the individual variation in timing of arrival, and the time spent by foxes at hotspots. Second, by analyzing their pattern of use of the sea ice and their participation in hotspots, we assess the detection range of arctic foxes.

## Methods

### (a) Study area

We worked in the south plain of Bylot Island (73° N, 80° W), which is part of Sirmilik National Park, Nunavut, Canada. The 600 km^2^ study area encompasses approximately 60 km of coastline and extends up to 15 km inland. The arctic fox is the main terrestrial predator of the area, feeding primarily on brown (*Lemmus sibiricus*) and collared lemmings (*Dicrostonyx groendilandicus*), but also on greater snow geese (*Chen caerulescens atlantica*) [32]. Arctic foxes are socially monogamous and family groups (three or more individuals) are very rare in our population [33]. Land–fast ice surrounds Bylot from late October to late July [34]. Arctic foxes in the area partly forage during winter on marine mammal carcasses from beached animals or kills left by polar bears (*Ursus maritimus*) [35]. They can prey on ringed seal (*Phoca hispida*) pups [28] when they become available in mid–March [36].

### (b) Capture and satellite tracking

As part of an ongoing study on arctic fox ecology, 6 to 26 adults were collared annually with Argos Platform Terminal Transmitters from 2007 to 2010 (KiwiSat 202, Sirtrack Ltd., Hawkes Bay, New Zealand; 95 g–115 g). Collars weighed 2.5–4.4 % of individuals’ body mass. We captured adults between May and August using padded leghold traps (Softcatch # 1, Oneida Victor Ltd., Euclid, OH, USA). If necessary, we anaesthetized animals through injection of medetomidine (0.05 mL/kg) and ketamine (0.03 mL/kg). We used atipemazole (0.05 mL/kg) as an antidote. All capture and handling of animals was approved by the appropriate authority and ethical committee (Université du Québec à Rimouski, permit # CPA32–08–62–R2). Field research was approved by the Joint Park Management Committee of Sirmilik National Park of Canada (permit # SNP–2009–2218).

We collared in 2010 the highest number of individuals (*n* = 26) since the beginning of the study. In addition, foxes foraged primarily on land during the winter 2010–2011, making relatively few excursions on the sea ice (see Results). Preliminary analyses showed that large sample size coupled with occasional extraterritorial movements provided ideal conditions to identify spatio–temporal fox hotspots, therefore we report here data from the winter 2010–2011. Collars transmitted from 14:00–17:00 UTC (08:00–11:00 local time) with a repetition rate of 60 s. Sixteen collars transmitted daily all year, while 10 others transmitted daily from 15 October to 15 May and every second day the rest of the year.

### (c) Spatial analyses

We filtered Argos locations with a speed filter implemented in R 3.2.0 (R Development Core Team). First, we kept only positions with a location class of LC 3, 2 and 1, respectively corresponding to errors < 250 m, between 250 and 500 m, and between 500 and 1500 m [37]. We then projected locations in the Universal Transverse Mercator*,* North American Datum 83 system and calculated the speed between successive locations. We removed any location requiring unrealistic speed values from the previous one (>7 km.h^−1^ speed, with possible 12–min acceleration bouts of 10 km.h^−1^). We set speed values from GPS data collected from the same population (D. Berteaux, unpublished data). After the removal of a location, the filter recalculated and evaluated again the speed between the new successive locations. We mapped locations using ArcMap 9.3 (ESRI, Redlands, CA, USA). The mean (± SD) number of locations per day per collar was 5.3 ± 2.1. We used locations from 25 October 2010 to 1 June 2011, starting from when the sea ice was completely formed around Bylot up to the beginning of cub rearing. Seven foxes dispersed during winter (see Additional file 1: Table S1) and thus moved completely out of the area. We excluded them from analyses starting from the day they left the study area.

We used the dynamic Brownian bridge movement model (dBBMM) implemented in the R package *move* [38] to estimate home ranges as well as individual– and population–level space use. The dBBMM combines the Brownian bridge movement model (BBMM) and the behavioral change point analysis to estimate the utilization distribution (UD) of an animal based on its movement path and a varying Brownian motion variance (σ^2^_m_) parameter that reflects changes in the movement behavior of the animal along the trajectory [39]. Like the BBMM, the dBBMM takes into account the elapsed time between consecutive locations (temporal autocorrelation) as well as the location error. The Brownian bridge approach is well suited for the study of mobile link species as it considers both the spatial and temporal aspects of movement [40]. To determine if a behavioral change occurred during the movement path, the dBBMM relies on a user–defined sliding window encompassing *w* locations along the path, and compares fit of models that use either one or two estimates of σ^2^_m_ for the window. Models using two estimates of σ^2^_m_ split the window in two parts at all possible breakpoints. The model with the lowest Bayesian Information Criterion (BIC) value is chosen. The sliding window produces several estimates of σ^2^_m_ for each segment, which are then averaged for the segment. The dBBMM requires a user–defined margin of at least 3 locations on each end of the window in which no breakpoint can be estimated [39]. The choice of the window and margin sizes should match the time interval within which behavioral changes are expected to occur [39]. Following this recommendation and after visual inspection of our data, we chose a window of 9 locations (corresponding to approximately 2 days) with a margin of 3 locations. We used the error radius provided by CLS [37], the company operating the Argos system, for each location.

**Fig. 1:**
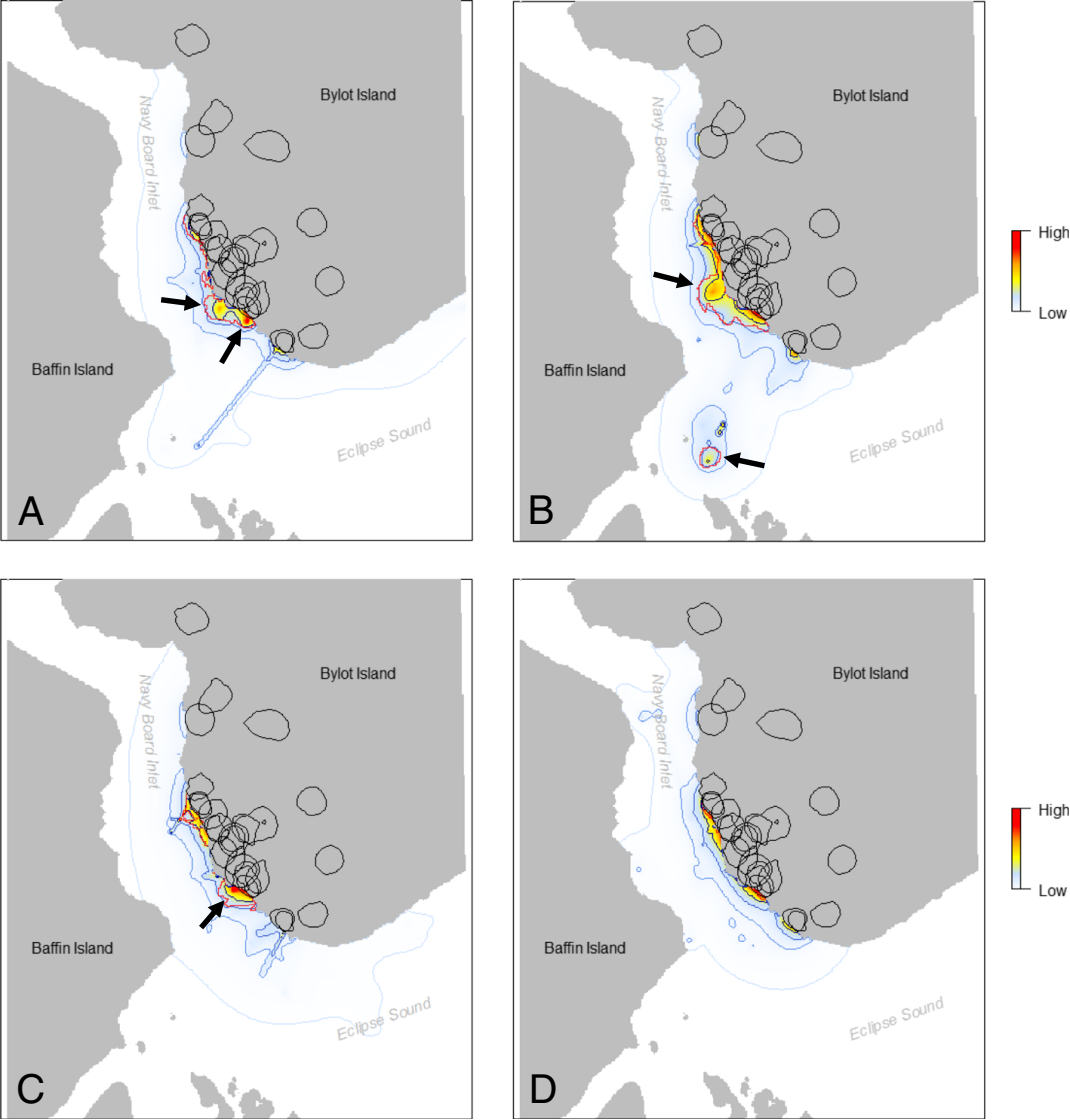
Estimated population–level use of the sea ice by arctic foxes using dynamic Brownian bridge movement models. Estimations for the month of **a** December, **b** January and **c** February, with black arrows indicating the spatio–temporal hotspots detected on the sea ice of Navy Board Inlet (Nunavut, Canada) during winter 2010–2011. The 25, 50, 75 and 99 % cumulative probability contours are shown in blue, with the darkest shades indicating the highest probabilities. Areas where more than 3 foxes occurred are delimited by a red line. Individual home ranges on Bylot Island are delimited by black lines. **d** Estimation for the month of November, when no hotspot was detected (shown for reference). Note that the coastline can appear as a relatively highly used area due to the back-and-forth crossing of foxes from their inland range to the sea ice, and to the home ranges located along the coast

We delineated the inland home range of each individual by calculating the dBBMM UD using locations on land and extracted the 50 % cumulative probability contours (core areas, Fig. 1). We then used dBBMMs to locate areas on the sea ice used intensively by foxes. Since the sea ice period covered more than 7 months, we divided it into smaller periods of 30 days to analyze sets of fox locations that were rather aggregated temporally. We used time slices of 30 days because carrion in cold climates can sometimes remain for at least a month during winter [12, 41]. Starting from 25 October 2010, we used a time window of 30 days moved in 2–week increments, so that time slices overlapped with each other. For each time slice, we calculated the dBBMM UD for each individual. We then summed the cell values of all individual UDs in order to obtain the population–level UD [42, 43]. Although arctic foxes can scavenge on and gather around terrestrial mammal carrion, such as caribou (*Rangifer tarandus*) or muskox (*Ovibos moschatus*) carcasses, we did not expect scavenging on land since there are no large herbivores in the study area. To facilitate visualization of the UD of the sea ice, we thus substracted *ad hoc* all land cell probabilities and re–scaled the resulting UD so that it summed to 1. One fox using repeatedly the same area sometimes led to high cell values in the population–level UD. In addition, pair mates foraging on the sea ice close to their home ranges also yielded high cell values along the coast (see Fig. 1d). For these reasons, we also calculated how many of the individual 75 % UDs (corresponding to the moderate to high–use areas) occurred within each cell of the population–level UD [43]. Resulting cell values ranged from 1 to n, with n ≤ the total number of individuals present during the analyzed time slice [43]. Finally, we identified highly–used areas visited by several foxes (“hotspots”), by selecting cells used by ≥ three foxes (Fig. 1).

**Fig. 2:**
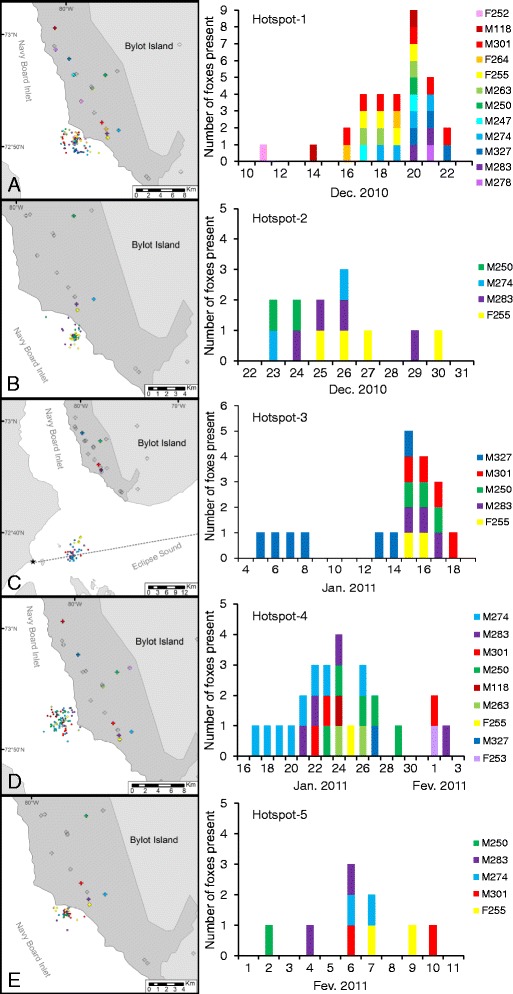
Fox locations for five spatio–temporal hotspots (**a-e**: Hotspot–1 to Hotspot–5) on the sea ice. Histograms show the chronology of arctic fox presence for each hotspot detected on the sea ice of Navy Board Inlet (Nunavut, Canada) during winter 2010–2011. Individual foxes are labeled with a letter (*M* for males and *F* for females) followed by their identity number. Crosses indicate the fox home range centers, with colored crosses for foxes detected at hotspots. The study area is depicted in dark grey. A star in (**c** ) shows where Pond Inlet hunters had stored some whale meat, with the dashed line indicating the straight route from the whale cache to Pond Inlet

Once hotspots were located, we identified individuals using each hotspot by intersecting fox locations on the sea ice with hotspot areas. We considered locations on the sea ice to be outside of fox home ranges if their distance to the home range boundary was higher than their associated Argos location error. The synchronous use of a given spot by several foxes is the most likely to reveal a carrion feeding event, thus we checked if foxes using a given area did so synchronously by analyzing the chronology of fox presence at the hotspots (Fig. 2). We kept only the hotpots where ≥ one fox was present during ≥ two consecutive days.

The thresholds used in our hotspot identification process are sometimes subjective, but they result in a conservative method most likely to identify carrion feeding events in a context where field validation was not possible due to the severe winter conditions of the High Arctic. Examples of hotspots that would have been selected through more liberal thresholds are shown in the Additional file 2: Figure S1.

We determined the center of each hotspot as the location with the highest UD value. For foxes visiting a hotspot, we calculated their distance from the hotspot as the distance they traveled between their last location the day before they joined the hotspot and the hotspot’s center, whether the foxes were in their tundra home range or out on the sea ice. For foxes not detected within a given hotspot, their distance to the hotspot was measured as their closest distance to the hotspot’s center during its existence.

### (d) Statistics

We used a mixed logistic regression to assess whether the probability that a fox joined a hotspot depended on its distance from it. Foxes that had not dispersed from Bylot Island were used as sampling units (*n* = 20–22 individuals per hotspot, for a total of 108 fox–hotspot pairs), with fox and hotspot identities included as random grouping variables. As a fox closer to the coast may be more aware of events occurring on the sea ice, we also assessed if the likelihood that a fox joined a hotspot depended on the distance of its home range center to the coastline. We compared each model with a null model using a likelihood–ratio test. We similarly assessed the relationship between the day of arrival of a fox at a hotspot and the length of its stay in this hotspot using a linear mixed effect model with foxes present at the hotspot as sampling units (*n* = 4–12 individuals per hotspot, for a total of 35 fox–hotspot pairs) and hotspot identity included as a random grouping variable. We could not add fox identity as a second random factor in this analysis because of the low sample size. This variable was however not significant (*L* = 1.86 × 10^−8^, *df* = 1, *p* = 0.99) when tested as a single random effect using a likelihood–ratio test and restricted maximum likelihood (REML) models [44]. Finally, we assessed if the distance that a fox traveled to a hotspot influenced the length of its stay at the hotspot, using the same procedure as when testing the effect of day of arrival. The number of days at the hotspot was log–transformed for this analysis to meet assumptions of normality and homogeneity of variances. We present summary statistics as means ± SD. We ran mixed models using the *nlme* [45] and *lme4* [46] libraries in R 3.2.0.

## Results

### (a) Information gathered on fox movements

The 26 tracked foxes (14 males and 12 females) yielded 47,634 locations which were reduced, after filtering, to 23,779 locations of LC 3, 2 or l (Additional file 1: Table S1). Seven foxes (5 males and 2 females) dispersed during winter and one female died. Twenty–four individuals were tracked during 2–7 months each (two collars failed in early November). From 25 October 2010 to 1 June 2011, these 24 foxes remained inland 85.8 ± 11.2 % (range: 65–100 %) of days tracked.

### (b) Spatio–temporal hotspots on the sea ice

**Fig. 3:**
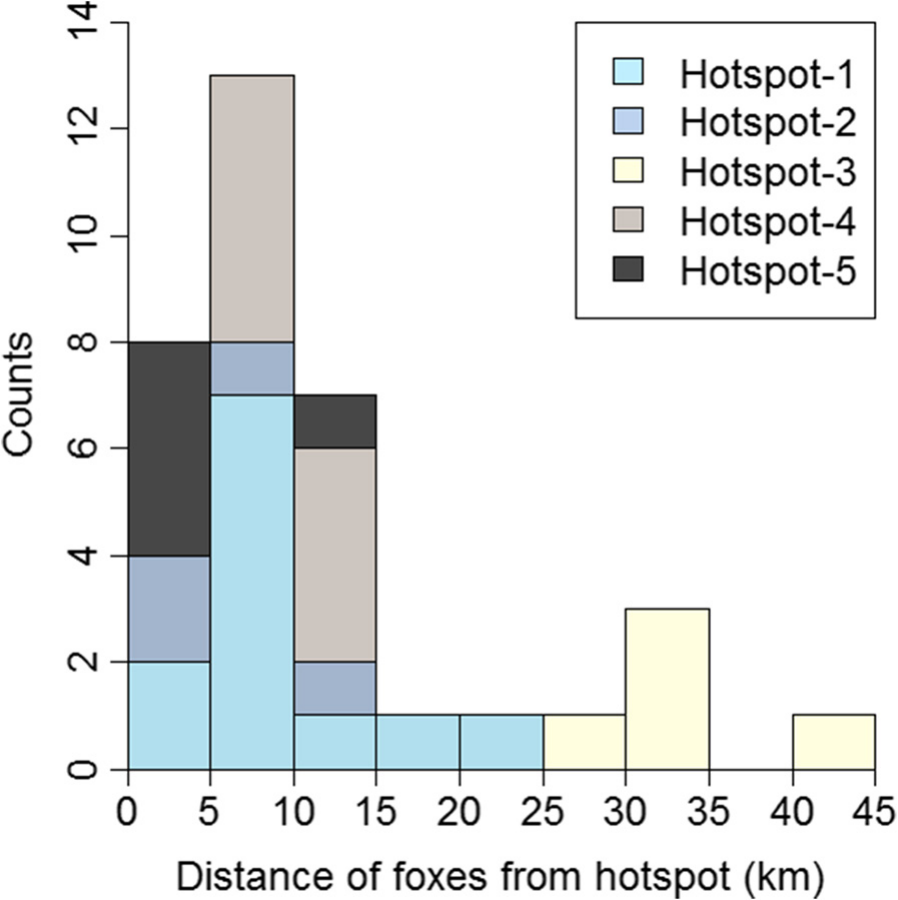
Frequency distribution of distances traveled by foxes to reach spatio–temporal hotspots on the sea ice. The 35 movements shown were performed by 13 arctic foxes moving to five spatio–temporal hotspots on the sea ice of Navy Board Inlet (Nunavut, Canada) during winter 2010–2011

Thirty–five movements to the sea ice by 13 individuals formed five spatio–temporal hotspots on the land–fast ice of Navy Board Inlet (Fig. 2). Four to 12 foxes visited a given hotspot, with a maximum of nine foxes present on the same day at Hotspot–1 (Fig. 2a). Foxes traveled on average 11.9 ± 9.9 km (range: 1.6–40.6 km, *n* = 35; Fig. 3) to join a hotspot. The maximum, 40.6 km travel distance, was recorded for a fox joining Hotspot–3 in January. The hotspots lasted 12 ± 3.3 days (range: 8–17 days) and individual foxes were present on average 2.8 ± 1.8 days at a given hotspot. The date of arrival and the minimum number of days spent at a site (1 to 8 days), however, varied considerably among individuals (Fig. 2). In 16 (45.7 %) of 35 instances when a fox joined a hotspot, the individual visited two or three times, with trips back to the territory between visits. Neither the distance of a fox to a hotspot, nor the distance between a fox territory and the coast influenced the likelihood of that fox joining the hotspot (respectively, coefficient −0.07, SE 0.05; χ^2^ = 1.48, *df* = 1, *p* = 0.22 and coefficient −0.43, SE 0.29; χ^2^ = 3.03, *df* = 1, *p* = 0.08). Foxes arriving late at a hotspot stayed fewer days than those arriving early. On average, a fox decreased the length of its stay at a hotspot by 1 day for every 4 days passing since the beginning of the hotspot (coefficient −0.23, SE 0.08; χ^2^ = 4.16, *df* = 1, *p* = 0.04). However, the distance a fox traveled to a hotspot did not influence the length of its stay (coefficient 0.01, SE 0.01; χ^2^ = 0.94, *df* = 1, *p* = 0.33).

### (c) Use of the sea ice by foxes joining hotspots

From October to mid–March (when carrion is the only substantial food available on the sea ice), the 13 foxes that were detected at least once at a hotspot remained inland 83.7 ± 9.1 % (range: 66.7–95.6 %) of the days they were tracked. These foxes were located at hotspots 35.2 ± 24.1 % of the days they used the sea ice. However, for nearly half of these foxes (6 out of 13), 44.8 to 75 % of their days spent on the sea ice were at a hotspot (Table 1). In addition, while the sea ice is already accessible in October, four individuals were first detected on the sea ice when visiting a hotspot (three foxes to Hotspot–1 in December and one to Hotspot–4 in January). Foxes were inside their home range before moving to a hotspot, except four times (out of 35) when they were already on the sea ice. In 30 times out of 35 (85.7 %), foxes were back inside their home range the day after leaving a hotspot (three were back 2 days after). The spatial and temporal dynamics of arctic foxes converging to hotspots is best illustrated through animated maps (see Additional file 3).

**Table 1:** Presence on land, on the sea ice, and at hotspots of 26 satellite–tracked arctic foxes

Fox ID	Proportion of days on land	Nb. of hotspot visited	Nb. of days on sea ice	Proportion of sea ice days at hotspots (Nb. of days)
M283^a^	85.9	4	20	75 % (15)
M263	94.2	2	8	62.5 % (5)
M274	78.7	4	26	61.5 % (16)
M250	83.6	5	23	52.2 % (12)
M301^a^	77.0	4	32	50.0 % (16)
F255	78.4	5	29	44.8 % (13)
M327	66.7	3	29	34.5 % (10)
F264^a^	94.9	1	7	28.6 % (2)
F253^a^	95.6	1	6	16.7 % (1)
M247	87.3	1	18	11.1 % (2)
M118	70.5	2	33	9.1 % (3)
F252	88.7	1	16	6.3 % (1)
M278	86.5	1	19	5.3 % (1)
M166	64.0	0	41	˗
F168	68.2	0	14	˗
F277	89.9	0	14	˗
F276	93.3	0	9	˗
F318	94.3	0	8	˗
M275	96.1	0	2	˗
F256	100	0	0	˗
F270	100	0	0	˗
F272	100	0	0	˗
F273	100	0	0	˗
M271	100	0	0	˗
M333	100	0	0	˗
M334	100	0	0	˗

Data range from 25 October 2010 to 15 March 2011, when the only substantial food source on the sea ice is carrion. Foxes that were present at hotspots are shown in the top half of the table, followed by those that were not. *F* females, *M* males

^a^indicates individuals for which the first presence on the sea ice was a visit to a hotspot

## Discussion

### (a) Attraction to hotspots and detection range

Satellite–tracked arctic foxes converged at specific areas on the sea ice of the Canadian Arctic. Individuals joining a hotspot usually left their inland home range to do so, and traveled back to it when they left the hotspot. These foxes remained mainly inland during winter (>80 % of the time), with some foxes present at identified hotspots during a relatively high proportion of the days they spent on the sea ice. In addition to the use of the sea ice by foxes being relatively low and often associated with visits to hotspots, four individuals left their inland home range for the first time of the winter to go to a hotspot. These results may indicate that foxes did not forage on the sea ice routinely during that winter, but rather used this habitat opportunistically, when the availability of carrion was detected, possibly from the home range. On these occasions, foxes traveled long distances to reach sea ice hotspots, often more than 10 km and up to 40 km. Altogether, the above suggests that foxes may be attracted to carrion from a relatively long distance. Note that although some fox trips on the sea ice appeared unrelated to any hotspot, not all foxes were collared in the area, and these trips could thus have led to undetected hotspots.

The dark and cold conditions prevailing during the study prevented us from sampling food sources on the sea ice, yet we can assume that food of marine origin was present. In addition, since all hotspots were detected from December–February, before seal pups were born, this food must have been carrion. Whereas all species of arctic whales leave the area before winter [47], ringed seals remain abundant all year round. They are fed upon by polar bears, which sometimes act as surplus killers in addition to often eating seals only partially [48–50]. Hunters from Pond Inlet, the closest Inuit community, also hunt seals, but they usually do not leave remains on the ice (C.–A. Gagnon, personal communication). A likely, testable hypothesis is therefore that hotspots occurred around ringed seal carrion left by polar bears. The size (several km^2^) of hotspots may be explained by a combination of 1– polar bears leaving clusters of carcasses rather than single carcasses, 2– foxes moving in the vicinity of carcasses between meals, and 3– Argos location error.

An alternative, non–mutually exclusive hypothesis may explain the specific case of Hotspot–3 (Fig. 2c). A few days before this hotspot was formed, Pond Inlet hunters transported by sledge, some bowhead whale meat that had been retrieved from a cache set up the previous summer; some meat may have been inadvertently lost on the ice (A. Maher, Parks Canada agency, personal communication).

### (b) Mechanisms involved in long–range food detection

The concurrent visit of the same areas by several individuals may indicate that they are guided by the same cues. Mammalian scavengers rely mostly on olfaction to find carcasses [18, 20], thus a logical hypothesis is that arctic foxes used long–range olfactory detection to detect carrion on the sea ice. The good olfactory capability of arctic foxes is well known as they can detect frozen lemmings under 46–77 cm of packed snow [27] or a subnivean seal lair (the excavated snow cavity made by a seal above a breathing hole) through snow depths of over 150 cm [28]. Our study may provide, however, the first estimates of long–range food detection for this species. It is noteworthy that marine mammal carcasses are very smelly and scent sources are rare on the sea ice, so that any new carrion may be readily detected. In addition, prevailing winter winds in Pond Inlet, located 60 km from our study area, are from the south and southwest [51], and could thus carry scents from the sea ice to fox territories (Fig. 1). On the other hand, the cold winter temperatures might hamper the generation and propagation of odors.

Among other arctic mammals, polar bears are notorious for their excellent sense of smell [49, 52]. Their scent detection distances vary from 2 to 3 km for a seal to 16 km for a large carcass [53] and even 60 km according to a popular publication [54], although the evidence is unclear in this last case. Unfortunately, olfaction–based detection distances have rarely been studied experimentally in mammals, and the few distances obtained through experiments [55, 56] are well below those reported here. Interestingly, however, observers following scent detection dogs (*Canis familiaris*) tracking seal lair or excavation [28, 57] or whale scats at sea [13] reported scent detection distances of up to 2–3 km. Some telemetry studies report higher distances, but with some caveats. For example, cattle carcass pits attracted resident and transient coyotes (*Canis latrans*) from 12.2 km and 20.5 km, respectively [58], but it is unclear whether coyotes detected carcasses remotely or were just revisiting productive sites. To our knowledge, the only strong evidence for a detection distance approaching estimates provided by our study is that of Nevitt et al*.* [16] who found through GPS tracking that the wandering albatross (*Diomedea exulans*) is capable of olfactory detection from over 20 km.

Other foraging tactics could be involved, such as the following of cues left by polar bears in the same way coyotes, ravens and red foxes (*Vulpes vulpes*) track wolf trails in the snow to find their kills [59–62], conspecific cueing mediated through chemical communication such as scent marks or scent trails as suggested for black bear (*Ursus americanus*) [63], coarse–level local enhancement [64] or inter–guild social information [65]. Until the nature of the items attracting foxes is clearly identified, some uncertainty remains about the method of detection and the exact activities of foxes on the sea ice, thus requiring further investigation. Moreover, some fox trips to the sea ice appeared unrelated to any hotspot. Foxes may thus also move onto the sea ice without *a priori* knowledge of food location. All of these hypotheses regarding long–range detection need testing and the arctic fox study system could offer productive avenues for experimental research, especially in late winter when light and temperature constraints are released in the Arctic. In particular, the experimental use of seal carcasses, coupled with Argos telemetry and camera traps [66], could yield new evidence. The use of tracking devices with finer spatial and temporal resolutions than Argos, such as GPS, would also allow a more precise estimation of detection distances, through e.g. detailed analysis of movement paths [16].

### (c) Behavioral and ecological implications of long–distance detection range

Because of the patchiness and unpredictability of marine resources, foraging on the sea ice is usually considered to be more risky for foxes than foraging on land [25, 67]. Our finding that marine resources may be detected from within the fox territories may challenge this view, at least when local conditions (distance of territories from the coast, presence of seal carcasses on the sea ice, direction of prevailing winds) make our results transferable. The large detection range of foxes may allow them to adopt a dual habitat selection strategy; they defend the inland territory that is essential for breeding and summer feeding, while occasionally traveling on the sea ice to feed upon marine resources when detected. Foxes closer to a hotspot were not more likely to move out of their ranges to feed on the sea ice than foxes located further away, indicating that some foxes may choose to remain on the land even if carrion is available. Foraging on the sea ice may indeed present other constraints, such as the competition with conspecifics at the carcass, the risks of interacting with a dominant species such as the polar bear, or simply the energetic costs of traveling. In addition, the prolonged absence of a territory holder may increase the risk of intruders settling in the territory, as seen in birds [68, 69]. In red foxes, territory takeover can occur from 3 to 8 days after the death of its owner [70–72]. The attachment to the home range of arctic foxes was also highlighted by the fact that individuals joining successive hotspots returned to their inland home ranges in between, instead of remaining on the sea ice. The distance of foxes to the coast also did not influence the probability to join a hotspot, showing that foxes with home ranges not located directly on the coast are also able to detect carrion. Hotspots lasted about 1–2 weeks. Foxes coming from further away from the hotspot did not stay longer than the ones from a closer range, but the last foxes arriving spent less time there than did the first ones arriving, indicating that while the resource found may offset the costs of travel, it was depleted relatively rapidly. Additional knowledge on the nature and availability of winter carcasses in the study area, as well as on potential scavenger competitors, is needed to untangle the costs and benefits of alternative winter foraging strategies.

In general, the lack of information on resource detection abilities could lead to erroneous conclusions in animal movement research. For example, a straight–line movement of an animal towards a resource can result either from goal–oriented navigation based on a cognitive map or from a discovery made using long–range detection. Distinguishing between these alternatives requires measuring detection distances for specific resources [7]. Our results also suggest that detection ranges may be underestimated for some mammalian scavengers, with implications regarding the appropriate spatial scale at which study results should be interpreted. Furthermore, a better knowledge of the detection ranges of various scavenger species would help to understand the sequence of exploitation of carrion by different competitor species, and hence the potential effect of such resource pulses on community ecology. Finally, fox hotspots also represent locations where many individuals are close to each other, thereby increasing the risks of transmission of diseases such as rabies [73]. Rabies is a contact disease whose epidemiology with arctic foxes is still largely unknown [74]. Knowing from how far away foxes in a population can be coming into contact can help in modeling the spatial spread of epidemic outbreaks.

## Conclusions

Our results, based on the most extensive set of satellite tracking data obtained to date on territorial arctic foxes, provide the first indication that this species may have a large food detection range that extends far beyond the boundaries of the territory. This study presents estimates of detection range useful for interpreting and modeling movements of this mobile predator. The ability to detect a food source from a long distance may shape foxes’ decisions to stay inland or move onto the sea ice during winter. As mobile links, foraging arctic foxes contribute to the transfer of resources from the marine to the terrestrial ecosystem [25, 31]. A large detection range enhances the efficiency of resource searches [8] and may thus intensify the exploitation of marine resources by a terrestrial predator, causing cascading effects on tundra community dynamics. In this context, it is important to consider the long–range food detection abilities of arctic foxes and other arctic predators.

## Additional files

Additional file 1: Table S1. Summary of the winter tracking records of 26 arctic foxes from Bylot Island, Nunavut, Canada. (PDF 120 kb) Additional file 2: Figure S1. Examples of hotspots not retained by our hotspot selection process. While selected spatio–temporal hotspots (more than one fox present at least two consecutive days) typically showed a high concentration of locations at their center and relatively high temporal synchrony, hotspots that were not selected show a more sequential use of the area or a less clustered pattern of fox locations. Histograms show the chronology of fox presence for each hotspot. Individual foxes are labeled with a letter (*M* for males and *F* for females) followed by their identity number. Crosses indicate fox home range centers, with colored crosses identifying foxes detected at hotspots. The study area is depicted in dark grey. (PDF 96 kb) Additional file 3:
**Animation of arctic fox movements.** Locations of tracked arctic foxes are shown from 25 October 2010 to 31 May 2011. For each day (indicated at the bottom left of maps), locations of each given fox are connected to locations of the same fox from the previous day, in order to show movement paths. Locations of fox hotspots are depicted by white ellipses and their names correspond to those in Fig. 2. The 40–km travel of individual M327 appears at 0:36. (MP4 20092 kb)
