# Supplementary material for: Spatio–temporal hotspots of satellite–tracked arctic foxes reveal a large detection range in a mammalian predator
Source: Mov Ecol. 2015 Nov 15;3:37. doi: 10.1186/s40462-015-0065-2 (PMC4644628; doi:10.1186/s40462-015-0065-2)
Supplement: Additional file 1: Table S1. — Summary of the winter tracking records of 26 arctic foxes from Bylot Island, Nunavut, Canada. (PDF 120 kb) [file 40462_2015_65_MOESM1_ESM.pdf]

Additional file 1:

Table S1. Summary of the winter tracking records of 26 arctic foxes from Bylot Island, Nunavut, Canada. *F* females, *M* males. †Track ending at the death of the animal, *track ending due to collar failure.

Fox ID	First location	Last location	Nb. days tracked	Nb. raw locations	Nb. filtered locations	Start of dispersal
F168	25 Oct 2010	1 Jun 2011	220	2224	1223	13 Dec 2010
F252	25 Oct 2010	9 May 2011*	197	1966	975	
F253	25 Oct 2010	1 Jun 2011	220	2298	1145	
F255	25 Oct 2010	25 May 2011	213	2207	1231	8 Mar 2011
F256	25 Oct 2010	1 Jun 2011	220	2220	1044	
F264	25 Oct 2010	1 Jun 2011	220	2174	1133	
F270	25 Oct 2010	2 Nov 2010*	9	96	66	
F272	25 Oct 2010	19 Jan 2011†	87	1235	375	
F273	25 Oct 2010	31 May 2011	219	2012	1052	
F276	25 Oct 2010	29 May 2011*	217	2117	959	
F277	25 Oct 2010	1 Jun 2011	220	2211	1177	
F318	25 Oct 2010	31 May 2011	219	2229	1152	
M118	25 Oct 2010	1 Jun 2011	220	2366	1427	14 Feb 2011
M166	26 Oct 2010	31 May 2011	218	1468	631	22 Mar 2011
M247	25 Oct 2010	1 Jun 2011	220	2397	1510	
M250	25 Oct 2010	30 Apr 2011	188	1729	955	19 Mar 2011
M263	25 Oct 2010	1 May 2011*	189	1939	788	
M271	25 Oct 2010	12 Nov 2010*	19	178	89	
M274	25 Oct 2010	8 Mar 2011†	135	1977	675	26 Feb 2011
M275	25 Oct 2010	16 Dec 2010*	53	510	284	
M278	25 Oct 2010	19 May 2011*	207	2193	1036	
M283	25 Oct 2010	1 Jun 2011	220	2351	1293	
M301	25 Oct 2010	1 Jun 2011	220	2226	977	
M327	25 Oct 2010	21 Feb 2011*	120	649	334	
M333	25 Oct 2010	1 Jun 2011	220	2365	1315	
M334	25 Oct 2010	31 May 2011	219	2297	933	27 Jan 2011
Mean			181.1	1832.1	914.6	
SD			66.7	701.4	399.2	
