# Supplementary figures and images for: Spatio–temporal hotspots of satellite–tracked arctic foxes reveal a large detection range in a mammalian predator
Source: Mov Ecol. 2015 Nov 15;3:37. doi: 10.1186/s40462-015-0065-2 (PMC4644628; doi:10.1186/s40462-015-0065-2)

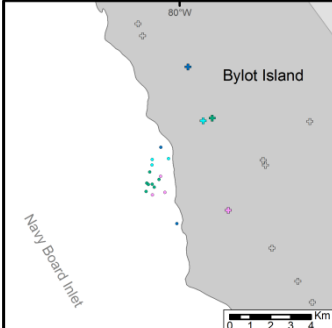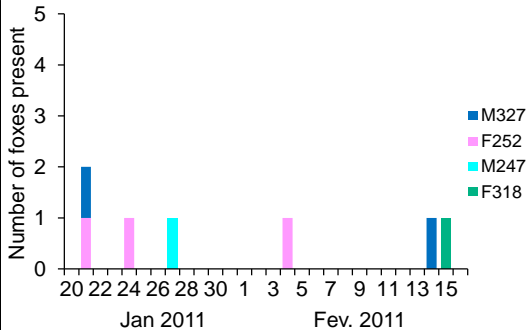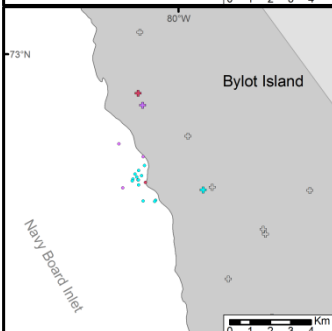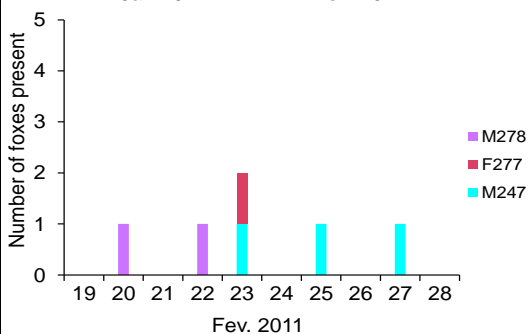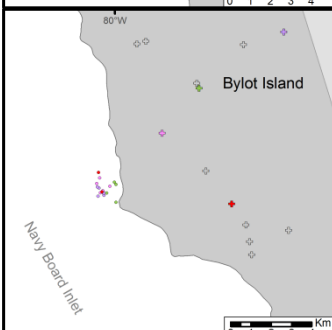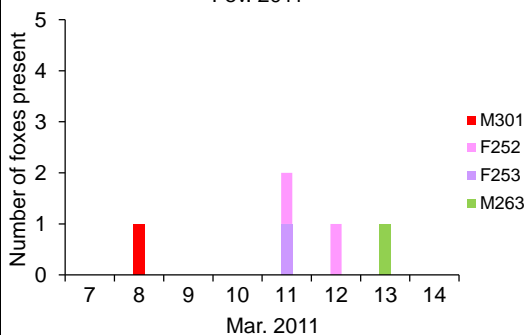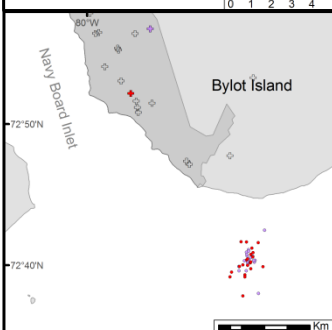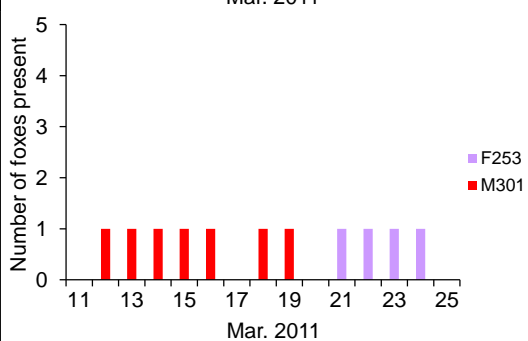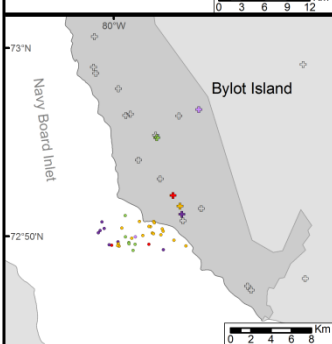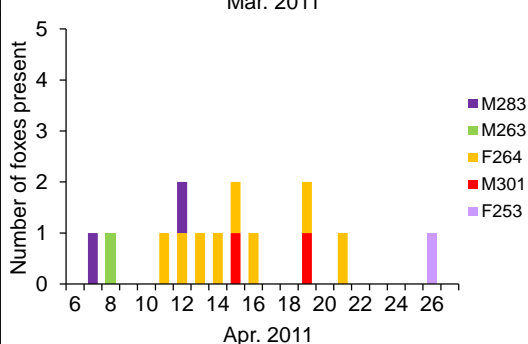

Supplement: Additional file 2: Figure S1. — Examples of hotspots not retained by our hotspot selection process. While selected spatio–temporal hotspots (more than one fox present at least two consecutive days) typically showed a high concentration of locations at their center and relatively high temporal synchrony, hotspots that were not selected show a more sequential use of the area or a less clustered pattern of fox locations. Histograms show the chronology of fox presence for each hotspot. Individual foxes are labeled with a letter (M for males and F for females) followed by their identity number. Crosses indicate fox home range centers, with colored crosses identifying foxes detected at hotspots. The study area is depicted in dark grey. (PDF 96 kb) [file 40462_2015_65_MOESM2_ESM.pdf]
